# Restoration of Upper Limb Function in an Individual with Cervical Spondylotic Myelopathy using Functional Electrical Stimulation Therapy: A Case Study

**DOI:** 10.3389/fneur.2016.00081

**Published:** 2016-06-10

**Authors:** Milos R. Popovic, Vera Zivanovic, Taufik A. Valiante

**Affiliations:** ^1^Institute of Biomaterials and Biomedical Engineering, University of Toronto, Toronto, ON, Canada; ^2^Rehabilitation Engineering Laboratory, Lyndhurst Centre, Toronto Rehabilitation Institute – University Health Network, Toronto, ON, Canada; ^3^Krembil Research Institute – University Health Network, Toronto, ON, Canada

**Keywords:** functional electrical stimulation, upper extremity rehabilitation, grasping, spinal cord disorders, myelopathy

## Abstract

Non-traumatic spinal cord pathology is responsible for 25–52% of all spinal cord lesions. Studies have revealed that spinal stenosis accounts for 16–21% of spinal cord injury (SCI) admissions. Impaired grips as well as slow unskilled hand and finger movements are the most common complaints in patients with spinal cord disorders, such as myelopathy secondary to cervical spondylosis. In the past, our team carried out couple of successful clinical trials, including two randomized control trials, showing that functional electrical stimulation therapy (FEST) can restore voluntary reaching and/or grasping function, in people with stroke and traumatic SCI. Motivated by this success, we decided to examine changes in the upper limb function following FEST in a patient who suffered loss of hand function due to myelopathy secondary to cervical spondylosis. The participant was a 61-year-old male who had C3–C7 posterior laminectomy and instrumented fusion for cervical myelopathy. The participant presented with progressive right hand weakness that resulted in his inability to voluntarily open and close the hand and to manipulate objects unilaterally with his right hand. The participant was enrolled in the study ~22 months following initial surgical intervention. Participant was assessed using Toronto Rehabilitation Institute’s Hand Function Test (TRI-HFT), Action Research Arm Test (ARAT), Functional Independence Measure (FIM), and Spinal Cord Independence Measure (SCIM). The pre–post differences in scores on all measures clearly demonstrated improvement in voluntary hand function following 15 1-h FEST sessions. The changes observed were meaningful and have resulted in substantial improvement in performance of activities of daily living. These results provide preliminary evidence that FEST has a potential to improve upper limb function in patients with non-traumatic SCI, such as myelopathy secondary to cervical spondylosis.

## Introduction

Although cervical spondylotic myelopathy (CSM) is categorized as a degenerative disorder most frequently seen in older individuals, this type of spinal cord dysfunction is influenced by genetic, environmental, and occupational factors as well. Hence, CSM affects a spectrum of patients across different age groups (1, 2), is the most common cause of spinal cord dysfunction in modern society worldwide (3), and is a major source of disability (4) that negatively affects quality of life (5, 6). Adults older than 55 years of age, who have cervical spondylosis are more predisposed to CSM as the aging process results in spondylotic changes, and these, in turn, result in direct compressive and/or ischemic dysfunction of the spinal cord (5, 6). The most characteristic CSM syndrome consists of varying combinations of the following triad: (a) painful stiff neck, (b) brachialgia and numb hands, and (c) spastic leg weakness with unsteadiness of gait (7). Upper extremity dysfunction is, in essence, one of the classic sign of CSM. This may have a variable presentation in the form of numbness or paresthesia, clumsiness, or weakness of the hand, hand and/or forearm muscle weakness, and atrophy, and occasionally there may be severe atrophy of the hand muscles. As a result, the individuals with CSM syndrome typically experience difficulty performing motor tasks, such as buttoning buttons, using keys, using a computer keyboard, pushing buttons on a cellular phone, or text messaging. These are considered as common early signs of CSM (8).

In a prospective study of CSM (8), it was observed that the earliest change indicative of CSM was the inability to adduct the little finger. This condition further progressed to affect the ring and middle fingers. As the disease became more severe, this loss of adduction in the fingers innervated by the ulnar nerve was followed by the inability to extend them (8). There are many underlying causes for this condition. In most cases, CSM is a progressive condition (9). Development of moderate to severe signs and symptoms of CSM usually indicate poor prognosis. In those cases in which the clinical status and imaging findings corroborate CSM diagnosis, surgical intervention may play a key role in managing CSM (9). Given the progressive nature of CSM, many clinicians advocate surgical treatment of patients with this condition.

In this article, we are presenting a case study in which functional electrical stimulation therapy (FEST) (explained in detail in the following section) was applied to an individual who had a CSM-related surgical treatment 22 months prior to taking part in the study. At the time, the participant enrolled in the study, he presented with progressive right hand weakness that resulted in his inability to voluntary open and close the hand and manipulate objects with his right hand. In what follows, we will present results that suggest that the FEST may be an effective intervention to manage CSM.

## Background

### Functional Electrical Stimulation

Functional electrical stimulation (FES) is a method that is able to produce functional movements in paralyzed muscles after an injury or damage to the central nervous system, including, but not limited to, stroke, spinal cord injury (SCI), and traumatic brain injury (10–19). By delivering low energy electrical pulses to nerves that are innervating muscles of interest (targeted muscles), the FES is able to induce controlled movements in the limbs and body (16, 18, 19). Careful application of highly controlled stimulation sequences is able to generate synergistic muscle activations that can produce complex movements, such as reaching, grasping, and walking.

### Functional Electrical Stimulation Therapy

Originally, FES was envisioned to be used as an orthotic device (also known as neuroprosthesis), intended to be worn permanently with users activating it whenever he/she required to reach or grasp an object, or to walk. Our team and others have been developing an alternative method for using FES technology. In this embodiment, FES technology is used to deliver a short-term therapeutic intervention, where the patient is expected to undergo a finite number of FEST sessions. Upon completion of the FEST program, the patient will have recovered partial or complete voluntary motor function in the targeted extremity, i.e., upper or lower extremity (20–30). The FEST program is designed to “retrain” the injured neuromuscular system, through repetitive performance of task-specific exercises. The FES during these training sessions is used to provide assistance with the components of the task that the individual is unable to perform independently. The assistance provided by the FES system to accomplish each task during the training session is determined based on day-to-day performance. At the completion of the 40–60 1-h-long FEST sessions, the individual is usually able to perform the tasks unassisted or with minimal assistance (i.e., without the help of FES device).

We have successfully used our FEST program to assist (i) adults with incomplete and complete SCI, and (ii) adults and children with severe upper limb deficit following stroke, to recover sustained reaching and/or grasping motor functions (22, 24, 25, 27–30). In our upper limb FEST program, the participant must attempt to initiate or execute the specific motor task unassisted, such as pinch grasp. Once a brief (10- to 20-s long) attempt to perform the specific task has been made, the therapist delivers FES-induced electrical pulses to the muscles to assist the individual to complete the task. Multitudes of different reaching and grasping tasks are trained. Each task is slightly different, and each task is trained on average between 5 and 7 min. During the early stages of FEST, performance of the entire task may be supported by FES, if required. As the therapy progresses, FES assistance is slowly reduced and eventually phased out. We believe that the combination of (i) active participation of the patient during therapy, (ii) the way in which FES system generates the movement, (iii) the fidelity of the movement performed using FES, (iv) the accuracy with which FES system mimics the natural limb movements, and (v) repetitive FES-induced movements are critical ingredients of this therapy. Previous experiments in SCI and stroke populations, conducted by our team (2, 22–24, 26–28, 30–32) and others (18, 33–35), indicate that improvements in grasping function, as a result of FEST, are meaningful and clinically relevant.

Currently, the mechanisms responsible for recovery of function using FEST are unknown. However, few physical and neurological mechanisms that may explain the effects of this therapeutic intervention have been already identified. The “peripheral” mechanisms are (i) muscle strengthening, (ii) increased flexibility and range of motion in the affected limb, and (iii) reduced muscle spasticity. The “neurological” mechanisms are (i) cortical reorganization, (ii) neuroplastic changes in the central nervous system, (iii) strengthening of the synaptic connection *via* Hebb’s rule, and (iv) functional reorganization and retraining of unaffected, but functionally related, areas of the central nervous system, allowing them to take control over the damaged parts of the central nervous system. Presently, the FEST experts are inclined to explain the improvements using the above listed and few additional neurological mechanisms (36).

### Motivation for This Study

In conclusion, there is strong evidence to support the use of FEST as an effective tool for retraining of reaching and grasping functions in neurological populations, such as stroke or SCI. Since CSM is a degenerative disorder of the cervical spine, which can be grouped among non-traumatic SCIs, it is very likely that the FEST for reaching and grasping will help this patient population improve voluntary reaching and grasping. Furthermore, it has been shown that strengthening muscles with the help of FES may as well bring about considerable improvements in walking and standing functions in the individuals with CSM (37). Therefore, this study was conducted to test if similar outcomes can be achieved in individuals with CSM following FEST. To the best of our knowledge, this is the first reported case of a chronic CSM patient treated with FEST for grasping.

## Discussion

### Diagnosis

The study participant was a 61-year-old male who underwent C3–C7 posterior laminectomy and instrumented fusion in November 2012, for cervical myelopathy. Cervical spine X-ray showed degenerative changes more prominent at C4–C7 levels. Following surgery, the participant experienced progressive weakness in the right hand. In November 2013, he underwent a redo cervical decompression and extension of fusion down to T2 level. Cervical spine MRI revealed moderate foraminal stenosis at C5–T1, and it was recommended that he undergoes re-exploration of his previous fusion and extension down to T2, bilateral foraminotomies, at C5–C6, C6–C7, and C7–T1. Postoperatively, the study participant developed right upper extremity numbness and progression of hand weakness. A cervical spine MRI was obtained demonstrating no acute findings or evidence of epidural hematoma and otherwise expected postoperative findings. The numbness and progression of hand weakness were thought to be related to intraoperative nerve root manipulation, which are typically transient in nature. At 22 months post initial surgery, the study participant continued to have right upper extremity numbness and weakness of the hand, as well as wasting of the intrinsic muscles of the right hand. The participant was unable to extend the lateral three fingers, namely, the middle, ring, and little fingers, despite relatively well-preserved function of the wrist. He was also unable to perform rapid extension of the fingers.

Typically, following the surgery for CSM, one can expect significant functional recovery, which should plateau at 6 months following the intervention (5, 6). Since the patient did not experience any significant improvement in hand function at 22-month mark, and since his hand function continued to deteriorate, he was invited to participate in this FEST case study.

### Treatment

The patient was screened to confirm that he did not have any contraindications (i.e., pacemaker, spinal stimulation, rash or open wound at any potential electrode site) to FEST. Prior to participating in the study, FEST was explained to the patient, including the risks and benefits of this treatment. After he received all pertinent information, the patient signed an informed consent form that was approved by the Research Ethics Board of the Toronto Rehabilitation Institute – University Health Network.

Our team has developed a hardware platform that can be used to deliver the FEST for improving upper limb function. This system is called Compex Motion (38), and it has four current regulated stimulation channels. The system can be used to deliver customized and diverse reaching and grasping FES protocols. In this particular case, the participant received FES protocols that enabled him to perform hand opening, palmar grasp, and lateral pinch grasp. The stimulation was delivered using standard self-adhesive surface stimulation electrodes, which were placed on the subject’s skin above the following muscles:

- flexor digitorum superficialis muscle and the flexor digitorum profundus muscle (finger flexion);
- flexor pollicis brevis (thumb flexion);
- extensor digitorum muscle (finger extension);
- lumbrical muscles (finger extension).

Stimulation parameters used to stimulate the above muscles were

- balanced, biphasic, current regulated electrical pulses;
- pulse amplitude from 6 to 30 mA (muscle dependent);
- pulse duration from 0 to 250 μs;
- pulse frequency 40 Hz.

The stimulation sequences (protocols) for this patient were tailored to his clinical presentation and his goals of therapy. The protocols allowed the participant to grasp and manipulate objects with the help of FES delivered by the Compex Motion system. The command for activating the stimulation sequence was initiated by a pushbutton that was triggered by the therapist who administered the therapy. Typical activation process was as follows. The participant was instructed on a particular grasping or hand opening task that he was supposed to perform. Then, the therapist instructed the participant to try to perform that task. The participant was given 10–20 s to try to execute the task on his own. Only after the participant completed as much of the task as he could by himself, the FES was triggered by the therapist to assist the participant in completing the entire task/movement. The participant received FEST three times per week for 5 weeks, i.e., 15 treatments sessions in total.

The subject was trained to perform (i) hand opening using finger extensors only, (ii) hand opening using a combination of lumbrical muscles and finger extensors, (iii) palmar grasp, and (iv) lateral pinch grasp. Throughout FEST sessions, the participant was taught how to approach, reach, grasp, and manipulate various objects without a fear that he will drop them during the object manipulation process. The FES-assisted grasping ensured that all objects were grasped robustly and could be manipulated with ease. With time, this training allowed the participant to become more confident in reaching, grasping, and manipulating objects. As a result, with time, the participant started to perform the same reaching, grasping, and object manipulation tasks, without using FES.

### Outcome Measures

At baseline and following completion of 15 FEST sessions, the participant was assessed using the following standardized assessments. The assessments were performed by a researcher who was not involved in the therapy delivery process.

#### Toronto Rehabilitation Institute’s Hand Function Test

Toronto Rehabilitation Institute’s Hand Function Test (TRI-HFT) (39) was used to assess unilateral gross motor function of the hand. The TRI-HFT consists of two sub-tests. The first one evaluates object manipulation skills using palmar grasp and lateral or pulp pinch grasp, and the second one evaluates the strength of both palmar and lateral pinch grasps. Note: TRI-HFT has been found to be very responsive to FEST.

#### Action Research Arm Test

Action Research Arm Test (ARAT) (40, 41) is a test developed to assess the upper limb function in stroke patients, and it consists of four sub-tests: grasp, grip, pinch, and gross movement.

#### Functional Independence Measure

Functional Independence Measure (FIM) (42) is an assessment specially developed to measure the degree of independence. The FIM is commonly used by both rehabilitation institutions and insurance companies to monitor patients’ improvements during rehabilitation.

#### Spinal Cord Independence Measure

Spinal Cord Independence Measure (SCIM) (43) is a disability scale that has been specifically developed to evaluate the functional outcomes of patients with traumatic and non-traumatic SCI.

## Results

**Figure 1:**
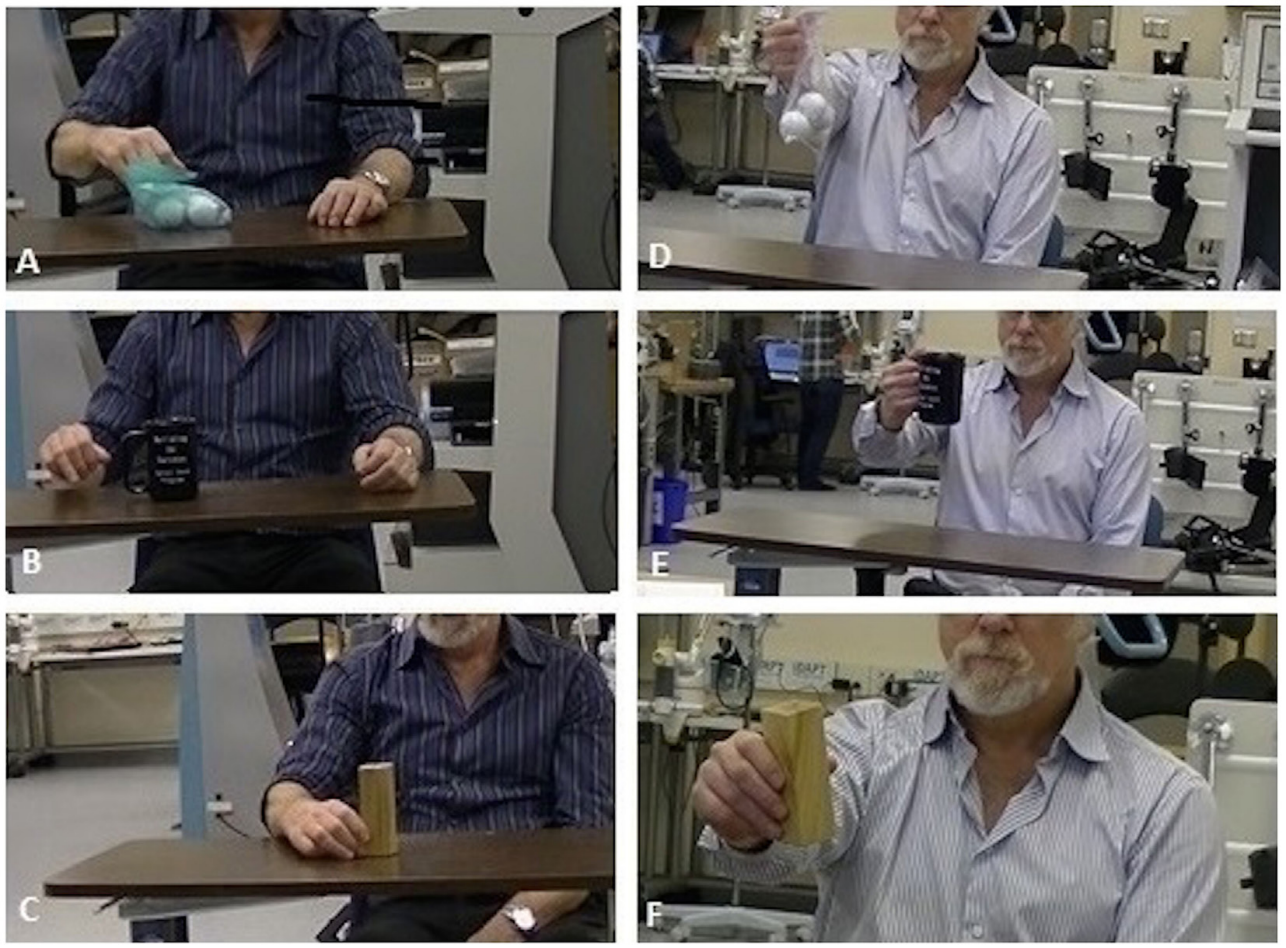
**TRI Hand Function Test – object manipulation**. **(A–C)** Object manipulation before FES therapy, i.e., patient unable to grasp and manipulate objects, and **(D–F)** Object manipulation after FES therapy, i.e., patient able to grasp and manipulate objects.

The participant completed 15 therapy sessions, as well as baseline and discharge assessments. Following 5 weeks of FEST, the participant was able to pick up small objects, which he was unable to do prior to FEST (see Figure 1). Table 1 summarizes all the clinical assessment scores at baseline and at discharge (i.e., after completion of therapy).

**Table 1:** **Participant’s scores on all outcome measures at baseline and at discharge**.

Outcome measures	Scores		
	Baseline	Discharge	Change
**ARAT**			
• ARAT – total score (MCID = 5.7)	37	44	7^a^
• ARAT – grasp	13	17	4
• ARAT – grip	10	10	0
• ARAT – pinch	5	8	3
• ARAT – gross movement	9	9	0
**TRI-HFT**			
• TRI-HFT – object manipulation (MCID not available)	54	65	11^a^
• TRI-HFT – wooden blocks (MCID not available)	48	62	14^a^
• TRI-HFT – instrumented cylinder (Nm)	0.2	0.5	0.3
• TRI-HFT – credit card (N)	0	5	5
• TRI-HFT – wooden bar thumb direction	3	10	7
• TRI-HFT – wooden bar little finger direction	30	30	0
**FIM**			
• FIM – total score (MCID = 22)	109	119	10
• FIM – self-care sub-score (MCID not available)	25	35	10
**SCIM**			
• SCIM – total score (MCID not available)	92	95	3
• SCIM – self-care sub-score (MCID not available)	12	15	3

*ARAT, Action Research Arm Test; TRI-HFT, Toronto Rehabilitation Institute’s Hand Function Test; FIM, Functional Independence Measure; SCIM, Spinal Cord Independence Measure; MCID, minimum clinically important difference*.

*^a^Change scores represent clinically meaningful change*.

This case study examined the efficacy of FEST as a clinical intervention for treating severe upper extremity impairment due to myelopathy secondary to cervical spondylosis. We found that the participant experienced considerable and clinically meaningful improvements in hand function as measured by TRI-HFT and ARAT. The pre/post differences in scores on all measures clearly demonstrated improvement in voluntary hand function following FEST. It is noteworthy that, in subacute stroke and traumatic SCI individuals, at least 20 and preferably 40 1-h FEST sessions are required to achieve clinically meaningful change. The fact that we were able to elicit clinically meaningful change in a chronic patient with CSM after only 15 1-h sessions suggests that the FEST for upper limb may be a viable therapeutic intervention for this patient population. These results provide preliminary evidence that rehabilitation treatment consisting of repetitive FEST designed to improve arm and hand function has the potential to restore voluntary grasping function in patients with CSM. The results also suggest that FEST leads to subsequent improvements in the quality and complexity of tasks that the participant was able to execute before and after study completion. These improvements in hand function subsequently lead to improvements in activities of daily living and increased level of independence. Following therapy completion, the participant subjectively reported (i) that he has observed improvements in his arm and hand function, and that he felt that he gained additional independence in activities of daily living; (ii) that the FEST allowed him to “awake his hand” when his fingers felt “dead” and “glued together”; (iii) that he experienced “more normal” movements in his hand and greater articulation in his fingers especially with respect to fine motor skills; and (iv) that he is now functioning at a “higher level.”

## Concluding Remarks

The results of our study are very encouraging; however, the study has certain limitations. This is a single case study with no control group, which makes it difficult to comment on comparative improvement that could have been achieved if the participant was administered a same dose of conventional occupational therapy alone. However, we have to acknowledge that the participant decided to participate in this study because he had already reached a plateau in recovery following many sessions of conventional occupational therapy. Based on the results of this case study, we believe that a study with a larger number of CSM patients is warranted, where the effects of FEST for reaching and grasping can be compared against current best practices for treating this patient population.

## Author Contributions

The work presented here was carried out in collaboration between all authors: MP, VZ, and TV. MP contributed to the design of the study, interpreted the results, and wrote the paper. VZ co-worked on associated data collection, interpreted the results, and wrote the paper. TV co-worked on data interpretation and revising paper for important intellectual content. All authors have seen and approved the manuscript.

## Conflict of Interest Statement

Dr. MP cofounded the company MyndTec, Inc. (www.myndtec.com), which is presently manufacturing the product MyndMove^®^. MyndMove^®^ delivers Dr. MP’s multichannel FEST for reaching and grasping for stroke and SCI patients. Although Dr. MP is the Chief Technology Officer of the company, the company is operating independently from his research program at the University of Toronto and Toronto Rehabilitation Institute – University Health Network. The study was conducted independently of MyndTec, Inc. MyndTec, Inc. did not participate in the study design and execution.

## References

[1] Henderson FC Geddes JF Vaccaro AR Woodard E Berry KJ Benzel EC. Stretch-associated injury in cervical spondylotic myelopathy: new concept and review. Neurosurgery (2005) 56(5):1101–13. 10.1227/01.NEU.0000157929.85251.7C 15854260

[2] Law MD Bernhardt M White AA 3rd Cervical spondylotic myelopathy: a review of surgical indications and decision making. Yale J Biol Med (1993) 66:165–77. 8209553 PMC2588867

[3] Wu JC Ko CC Yen YS Huang WC Chen YC Liu L Epidemiology of cervical spondylotic myelopathy and its risk of causing spinal cord injury: a national cohort study. Neurosurg Focus (2013) 35:E10. 10.3171/2013.4.FOCUS13122 23815246

[4] Chang V Lu D Hoffman H Buchanan C Langston TH. Clinical results of cervical laminectomy and fusion for the treatment of cervical spondylotic myelopathy in 58 consecutive patients. Surg Neurol Int (2014) 5(Suppl 3):S133–7. 10.4103/2152-7806.130670 24843810 PMC4023005

[5] Furlan JC Kalsi-Ryan S Kailaya-Vasan A Massicotte E Fehlings M. Functional and clinical outcomes following surgical treatment in patients with cervical spondylotic myelopathy: a prospective study of 81 cases. J Neurosurg Spine (2011) 14:348–55. 10.3171/2010.10.SPINE091029 21235299

[6] Prabhu KL Babu KS Samuel S Chacko AG. Rapid opening and closing of the hand as a measure of early neurologic recovery in the upper extremity after surgery for cervical spondylotic myelopathy. Arch Phys Med Rehabil (2005) 86(1):105–8. 10.1016/j.apmr.2004.01.037 15640999

[7] Victor M Ropper AH Adams and Victor’s Principles of Neurology. 7th ed New York, NY: McGraw-Hill (2001).

[8] Ono K Ebara S Fuji T Yonenobu K Fujiwara K Yamashita K. Myelopathy hand: new clinical signs of cervical cord damage. J Bone Joint Surgery Br (1987) 69:215–9. 3818752 10.1302/0301-620X.69B2.3818752

[9] Harrop JS Naroji S Maltenfort M Anderson DG Albert T Ratliff JK Cervical myelopathy: a clinical and radiographic evaluation and correlation to cervical spondylotic myelopathy. Spine (2010) 35:620–4. 10.1097/BRS.0b013e3181b723af 20150835

[10] Biering-Sørensen F Hansen B Lee BSB. Non-pharmacological treatment and prevention of bone loss after spinal cord injury: a systematic review. Spinal Cord (2009) 47(7):508–18. 10.1038/sc.2008.177 19172152

[11] Groah SL Lichy AM Libin AV Ljungberg I. Intensive electrical stimulation attenuates femoral bone loss in acute spinal cord injury. PM R (2010) 2(12):1080–7. 10.1016/j.pmrj.2010.08.003 21145519

[12] Gyawali S Solis L Chong SL Curtis C Seres P Kornelsen I Intermittent electrical stimulation redistributes pressure and promotes tissue oxygenation in loaded muscles of individuals with spinal cord injury. J Appl Physiol (2011) 110(1):246–55. 10.1152/japplphysiol.00661.2010 20884840

[13] Kennelly MJ Bennett ME Grill WM Grill JH Boggs JW. Electrical stimulation of the urethra evokes bladder contractions and emptying in spinal cord injury men: case studies. J Spinal Cord Med (2011) 34(3):315–21. 10.1179/2045772311Y.0000000012 21756572 PMC3127369

[14] Kutzenberger J Domurath B Sauerwein D. Spastic bladder and spinal cord injury: seventeen years of experience with sacral deafferentation and implantation of an anterior root stimulator. Artif Organs (2005) 29(3):239–41. 10.1111/j.1525-1594.2005.29043.x 15725225

[15] Lai C-H Chang WH-S Chan WP Peng C-W Shen L-K Chen J-JJ Effects of functional electrical stimulation cycling exercise on bone mineral density loss in the early stages of spinal cord injury. J Rehabil Med (2010) 42(2):150–4. 10.2340/16501977-0499 20140411

[16] Masani K Popovic MR Functional electrical stimulation: applications in rehabilitation and neurorehabilitation. In: Kramme R Hoffmann K-P Pozos RS, editors. Handbook of Medical Technology. Berlin: Springer Handbooks (2011). p. 877–96.

[17] Minassian K Hofstoetter U Tansey K Mayr W. Neuromodulation of lower limb motor control in restorative neurology. Clin Neurol Neurosurg (2012) 114(5):489–97. 10.1016/j.clineuro.2012.03.013 22464657 PMC3341569

[18] Popovic DB Popovic MB Sinkjaer T Stefanovic A Schwirtlich L. Therapy of paretic arm in hemiplegic subjects augmented with a neural prosthesis: a cross-over study. Can J Physiol Pharmacol (2004) 82(8–9):749–56. 10.1139/y04-057 15523532

[19] Popovic MR Thrasher TA Neuroprostheses. In: Wnek GE Bowling GL, editors. Encyclopedia of Biomaterials and Biomedical Engineering. 2nd ed Marcel Dekker Inc. (2004). p. 1056–65.

[20] Giangregorio L Craven BC Richards K Kapadia N Hitzig SL Masani K A randomized trial of functional electrical stimulation for walking in incomplete spinal cord injury: effects on body composition. J Spinal Cord Med (2012) 35(5):351–60. 10.1179/2045772312Y.0000000041 23031172 PMC3459564

[21] Hitzig SL Craven BC Panjwani A Kapadia N Giangregorio LM Richards K A randomized trial of functional electrical stimulation therapy for walking in incomplete spinal cord injury: effects on quality of life and community participation. Top Spinal Cord Inj Rehabil (2013) 19(4):245–58. 10.1310/sci1904-245 24244090 PMC3816719

[22] Kapadia N Zivanovic V Furlan J Craven BC McGillivray C Popovic MR Toronto Rehabilitation Institute’s functional electrical stimulation therapy for grasping in traumatic incomplete spinal cord injury: randomized control trial. Artif Organs (2011) 35(3):212–6. 10.1111/j.1525-1594.2011.01216.x 21401662

[23] Kapadia N Popovic MR Toronto Rehabilitation Institute’s function electrical stimulation therapy for grasping in SCI: an overview. Top Spinal Cord Inj Rehabil (2011) 17(1):70–6. 10.1310/sci1701-70

[24] Kapadia N Zivanovic V Popovic MR. Restoring voluntary grasping function in individuals with incomplete chronic spinal cord injury: pilot study. Top Spinal Cord Inj Rehabil (2013) 19(4):279–87. 10.1310/sci1904-279 24244093 PMC3816722

[25] Kapadia N Nagai MK Zivanovic V Bernstein J Woodhouse J Rumney P Functional electrical stimulation therapy for recovery of reaching and grasping in severe chronic paediatric stroke patients. J Child Neurol (2014) 29(4):493–9. 10.1177/0883073813484088 23584687

[26] Kapadia N Masani K Craven BC Giangregorio LM Hitzig SL Richards K A randomized trial of functional electrical stimulation for walking in incomplete spinal cord injury: effects on walking competency. J Spinal Cord Med (2014) 37(5):511–24. 10.1179/2045772314Y.0000000263 25229735 PMC4166186

[27] Kawashima N Popovic MR Zivanovic V Effect of intensive functional electrical stimulation therapy on the upper limb motor recovery after stroke: single case study of a chronic stroke patient. Physiother Canada (2013) 65(1):20–8. 10.3138/ptc.2011-36 PMC3563372 24381377

[28] Popovic MR Thrasher TA Adams ME Takes V Zivanovic V Tonack MI. Functional electrical therapy: retraining grasping in spinal cord injury. Spinal Cord (2006) 44(3):143–51. 10.1038/sj.sc.3101822 16130018

[29] Popovic MR Kapadia N Zivanovic V Furlan JC Craven BC McGillivray C. Functional electrical stimulation therapy of voluntary grasping versus only conventional rehabilitation for patients with subacute incomplete tetraplegia: a randomized clinical trial. Neurorehabil Neural Repair (2011) 25(5):433–42. 10.1177/1545968310392924 21304020

[30] Thrasher TA Zivanovic V McIlroy W Popovic MR. Rehabilitation of reaching and grasping function in severe hemiplegic patients using functional electrical stimulation therapy. Neurorehabil Neural Repair (2008) 22(6):706–14. 10.1177/1545968308317436 18971385

[31] Miller RC Popovic MR Thrasher TA Verrier M Functional electrical stimulation therapy improves grasping in chronic cervical spinal cord injury: two case studies. J Automat Contr (2008) 18(2):53–62. 10.2298/JAC0802053M

[32] Popovic MR Thrasher TA Zivanovic V Takaki J Hajek V Neuroprosthesis for restoring reaching and grasping functions in severe hemiplegic patients. Neuromodulation (2005) 8(1):60–74. 10.1111/j.1094-7159.2005.05221.x 22151384

[33] Popovic D Stojanović A Pjanović A Radosavljević S Popovic M Jović S Clinical evaluation of the bionic glove. Arch Phys Med Rehabil (1999) 80(3):299–304. 10.1016/S0003-9993(99)90141-7 10084438

[34] Popovic MB Popovic DB Sinkjaer T Stefanovic A Schwirtlich L. Restitution of reaching and grasping promoted by functional electrical therapy. Artif Organs (2002) 26(3):271–5. 10.1046/j.1525-1594.2002.06950.x 11940031

[35] Prochazka A Gauthier M Wieler M Kenwell Z. The bionic glove: an electrical stimulator garment that provides controlled grasp and hand opening in quadriplegia. Arch Phys Med Rehabil (1997) 78(6):608–14. 10.1016/S0003-9993(97)90426-3 9196468

[36] Nagai MK Marquez-Chin C Popovic MR Why is functional electrical stimulation therapy capable of restoring motor function following severe injury to the central nervous system? In: Tuszynski MH, editor. Translational Neuroscience. New York, NY: Springer Science and Business Media LLC (2016). p. 479–98.

[37] Pastor D. Use of electrical stimulation and exercise to increase muscle strength in a patient after surgery for cervical spondylotic myelopathy. Physiother Theory Pract (2010) 26(2):134–42. 10.3109/09593980902750915 20067363

[38] Popovic MR Keller T. Modular transcutaneous functional electrical stimulation system. Med Eng Phys (2005) 27(1):81–92. 10.1016/j.medengphy.2004.08.016 15604009

[39] Kapadia N Zivanovic V Verrier M Popovic MR Toronto Rehabilitation Institute’s hand function test: assessment of gross motor function. Top Spinal Cord Inj Rehabil (2012) 18(2):167–86. 10.1310/sci1802-167 23459270 PMC3584758

[40] Carroll D A quantitative test of upper extremity function. J Chronic Dis (1965) 18:479–91. 10.1016/0021-9681(65)90030-5 14293031

[41] Lyle RC A performance test for assessment of upper limb function in physical rehabilitation treatment and research. Int J Rehabil Res (1981) 4(4):483–92. 10.1097/00004356-198112000-00001 7333761

[42] Dodds TA Martin DP Stolov WC Deyo RA. A validation of the functional independence measurement and its performance among rehabilitation inpatients. Arch Phys Med Rehabil (1993) 74:531–6. 10.1016/0003-9993(93)90119-U 8489365

[43] Catz A Itzkovich M Agranov E Ring H Tamir A SCIM – spinal cord independence measure: a new disability scale for patients with spinal cord lesions. Spinal Cord (1997) 35:850–6. 10.1038/sj.sc.3100504 9429264

